# Physical capacity of rescue personnel in the mining industry

**DOI:** 10.1186/1745-6673-3-22

**Published:** 2008-10-12

**Authors:** Ian B Stewart, Michael D McDonald, Andrew P Hunt, Tony W Parker

**Affiliations:** 1 Institute of Health and Biomedical Innovation, Queensland University of Technology, Brisbane, Australia; 2 School of Human Movement Studies, Queensland University of Technology, Brisbane, Australia

## Abstract

**Background:**

The mining industry has one of the highest occupational rates of serious injury and fatality. Mine staff involved with rescue operations are often required to respond to physically challenging situations. This paper describes the physical attributes of mining rescue personnel.

**Methods:**

91 rescue personnel (34 ± 8.6 yrs, 1.79 ± 0.07 m, 90 ± 15.0 kg) participating in the Queensland Mines Rescue Challenge completed a series of health-related and rescue-related fitness tasks. Health-related tasks comprised measurements of aerobic capacity (VO_2_max), abdominal endurance, abdominal strength, flexibility, lower back strength, leg strength, elbow flexion strength, shoulder strength, lower back endurance, and leg endurance. Rescue-related tasks comprised an incremental carry (IC), coal shovel (CS), and a hose drag (HD), completed in this order.

**Results:**

Cardiovascular (VO_2_max) and muscular endurance was average or below average compared with the general population. Isometric strength did not decline with age. The rescue-related tasks were all extremely demanding with heart rate responses averaging greater than 88% of age predicted maximal heart rates. Heart rate recovery responses were more discriminating than heart rates recorded during the tasks, indicating the hose drag as the most physically demanding of the tasks.

**Conclusion:**

Relying on actual rescues or mining related work to provide adequate training is generally insufficient to maintain, let alone increase, physical fitness. It is therefore recommended that standards of required physical fitness be developed and mines rescue personnel undergo regularly training (and assessment) in order to maintain these standards.

## Background

The mining industry has one of the highest occupational rates of serious injury and fatality throughout the world [1]. Mining accidents can have a variety of causes including leaks of poisonous gases, asphyxiant gases, dust explosions, collapsing mine stopes, flooding, or general mechanical errors from improperly used or malfunctioning mining equipment. Numerous accident scenarios can therefore develop that require specialist skills in handling hazardous materials, fires, search and rescue, vertical ascent, and vehicle accidents. The combination of the high incidence of accident with the multitude of possible accident scenarios requires that the mine staff who volunteer to be involved with rescue operations are commonly placed in both mentally and physically challenging situations.

In order to prepare for a rescue situation the mines rescue teams from within Queensland Australia, where mining represents a significant contributor to the gross domestic product and a large proportion of the workforce, undertake an annual event comprising a series of rescue simulations that challenge the teams in various aspects of mines rescue. The purpose of this paper was to describe the physical attributes of the mines rescue personnel and their physiological response to the simulated physical challenges that they may encounter during a rescue.

## Methods

### Participants

A total of 91 miners competing at the 2005 and 2006 mines rescue challenge were recruited to participate in this study. Subjects were fully informed of the experimental procedures prior to giving written consent to participate. Approval from the Queensland University of Technology Human Research Ethics Committee was obtained for this study.

### Health-related Fitness tests

Subjects completed a health screening questionnaire to ensure they were safe to participate. General descriptive information (age, height, & weight) were collected. Health-related fitness was measured by assessing the following attributes: aerobic capacity (VO_2_max), abdominal endurance, abdominal strength, flexibility, lower back strength, leg strength, elbow flexion strength, shoulder strength, lower back endurance, and leg endurance. The measurements were all conducted in an air-conditioned room.

VO_2 _max was estimated from a 6 minute step test. The subject stepped up and down a step height of 12" to the beat of a metronome. The first 3 minutes were at a pace of 15 steps per minute and the final 3 minutes were at 27 steps per minute. The heart rate from the final minute of each stage was applied to a linear regression with VO_2 _to extrapolate the data to the persons age predicted maximal heart rate, enabling an estimate of their VO_2_max [2]. Abdominal endurance was measured as the number of completed sit ups in 60 seconds [3]. Lower back endurance was assessed by the Biering-Sorensen test [4].

Maximal isometric strength was assessed with a customised strain gauge system linked to a computer program (LabVIEW, National Instruments, Austin, TX). The subjects performed a seated row, dead lift, standing shoulder press and bicep curl exercises. Force generated (kg) was obtained from a three second maximal effort. Abdominal strength was assessed as the number of different variations of sit up successfully completed. Seven different variations of sit up were used, each of an increasing difficulty. The subject attempted each one in order, until they could not complete a particular variation. The last successfully completed stage was recorded as their abdominal strength score [3]. Flexibility was assessed via the sit-and-reach test [5].

### Simulated Rescue Tasks

The simulated rescue tasks included an incremental carry (IC), coal shovel (CS), and a hose drag (HD), completed in this order. These tests had been previously validated as representative of work tasks in underground mining [6]. Each task lasted three minutes and the participant's heart rate was monitored by telemetry (s610i, Polar Oy, Finland) and averaged every five seconds for the duration of the challenge (approximately two hours). Subjects had adequate time (minimum of 24 minutes) for recovery between successive tasks. All simulated rescue tasks were completed outdoors in environmental conditions ranging from 20–26 degrees Celsius. The IC task required the subject to walk along a 40 m circuit (20 m out and 20 m back) whilst carrying a container, to which extra weights were added. The weight started at 5 kg, and was increased by 5 kg after completing each lap of the circuit, up to a maximum of 25 kg. The CS task involved a pit 2 m wide, 4 m long and 0.2 m high filled with coal. The length was divided in half by two 44 gallon drums (600 mm in diameter), lying end-on-end. The subject was required to stand in the pit and shovel the coal over the drums. The blade of the shovel was required to be covered in coal and all of the coal was required to travel over the barrels for the shovel to score. The total number of shovels completed in three minutes was counted. The first stage of the HD task required the subject to pull a 70 mm water hose wound around a drum, a distance of 10 m. Then the subject returned to the drum (walking), grasped the hose and pulled it 20 m. This process was repeated for 30, 40, and 50 m distances, or until the three minutes was completed.

### Statistical Analysis

All data presented are summarised as mean and standard deviation, unless otherwise specified. Participants were separated into age groups (20–29, 30–39, 40–49, & 50–59 years) to present the descriptive and health related fitness data. One-way Analysis of variance with Bonferoni post hoc tests were performed on all the health related fitness variables across the age groups. Repeated measures ANOVA (3 tasks × 3 time points) was used to assess the differences in heart rate recovery following the simulated rescue tasks.

## Results

A total of 79 subjects completed the health-related fitness tests, 27 of which also had their heart rate monitored throughout the simulated rescue tasks. An additional 12 subjects completed the simulated rescue tasks, but did not complete the health related fitness tests. Descriptive (Table 1) and health-related fitness characteristics (Table 2) of the subjects is provided. Participants aged between 40 – 49 years had a significantly lower VO_2 _max compared to those aged 30–39 years. Both abdominal strength and endurance were significantly lower in the 50–59 year age group in comparison to those 20–29 years of age. All other health-related fitness characteristics did not significantly differ across the age groups.

**Table 1:** Descriptive characteristics across the age groups

	**total**	**20–29**	**30–39**	**40–49**	**50–59**
	(n = 79)	(n = 28)	n = (31)	(n = 16)	(n = 4)
**Age (years)**	34 (8.6)	25 (2.6)	35 (2.5)	43 (2.3)	55 (1.5)
**Height (m)**	1.79 (0.07)	1.80 (0.08)	1.79 (0.08)	1.79 (0.04)	1.70 (0.05)*
**Weight (Kg)**	90 (15.0)	90 (16.9)	91 (14.86)	92 (11.2)	89 (21.7)
**BMI**	28 (3.8)	28 (4.0)	28 (3.6)	29 (3.2)	31 (6.7)

* significantly different from the 20–29 age group (p < 0.05)

All age groups significantly differ for age (p < 0.01).

**Table 2:** Health related Fitness characteristics across the age groups

	**Total**	**20–29**	**30–39**	**40–49**	**50–59**
	(n = 79)	(n = 28)	(n = 31)	(n = 16)	(n = 4)
*Endurance measures*					
**VO_2_max (ml/kg/min)**	42 (8.3)	42 (7.3)	44 (9.4)	37 (5.3) #	40 (11.9)
**Abdominal endurance (sit ups/60 sec)**	34 (8.8)	35 (7.9)	35 (8.0)	32 (10.6)	22 (9.0)*
**Lower back endurance (sec)**	117 (56.0)	122 (45.5)	123 (57.0)	97 (69.0)	107 (64.1)
					
*Strength measures*					
**Abdominal Strength (score 1–7)**	3.6 (1.7)	4.4 (1.6)	3.4 (1.5)	3.2 (1.8)	1.5 (0.6)*
**Bicep Curl (kg)**	44 (9.0)	42 (10.2)	45 (8.9)	45 (7.0)	37 (2.0)
**Shoulder Press (kg)**	66 (21.4)	71 (26.6)	70 (20.6)	63 (17.8)	48 (9.7)
**Seated Row (kg)**	134 (34.4)	134 (31.8)	141 (39.5)	127 (29.3)	106 (7.46)
**Dead Lift (kg)**	163 (46.2)	157 (39.7)	176 (55.6)	148 (35.8)	144 (22.7)
					
*Flexibility measures*					
**Sit & each (cm)**	6.5 (7.4)	5.3 (7.5)	8.3 (7.5)	5.4 (6.4)	3.8 (9.7)

* Significantly different from the 20 – 29 age group (p < 0.05)

# Significantly different from the 30–39 age group (p < 0.05)

**Figure 1:**
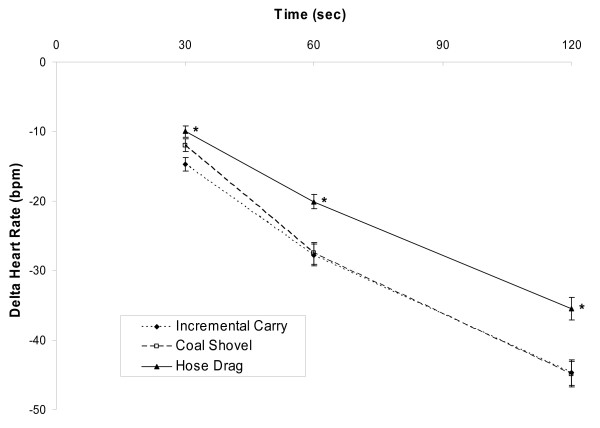
**Heart Rate Recovery following the three minute simulated rescue tasks (means ± SEM)**. * significantly different from incremental carry and coal shovel, p < 0.05.

The heart rate responses for the simulated rescue tasks are summarised in Table 3 and Figure 1. The incremental carry produced significantly lower average and peak heart rate responses during the task (Table 3), while the recovery heart rates following the hose drag was significantly higher compared with the other simulated rescue tasks (Figure 1). The time required to recover to 70% of the heart rate achieved during the task was significantly longer in the hose drag, than the coal shovel or the incremental carry (213 ± 14, 171 ± 10, 156 ± 10 seconds, respectively, p < 0.01).

**Table 3:** Heart Rate Response to Simulated Rescue Tasks

	**Average**		**Peak**	
	(bpm)	(% APMHR)	(bpm)	(% APMHR)
**Incremental Carry**	165 (10.3)*	88 (5.1)*	180 (10.5)*	97 (5.1)*
**Coal Shovel**	174 (10.0)	93 (4.6)	184 (9.4)	99 (4.0)
**Hose Drag**	174 (9.2)	93 (4.5)	183 (9.0)	98 (4.3)

NB. APMHR = age predicted maximal heart rate

* Significantly different from Coal Shovel and Hose Drag (p < 0.05)

## Discussion

Mining has historically been a physically demanding occupation, but with increased automation designed to increase productivity the perception has been that the physical nature of the job has been reduced. Recent analyses of work tasks at underground and open-cut mine sites has revealed that there are still numerous manual handling tasks that require significant levels of musculoskeletal strength and endurance [6]. Mines rescue personnel comprise volunteers from all occupations within the mining workforce, and as such they may or may not be exposed to physical demanding tasks while on the job. The level of physical training undertaken by the rescue personnel, both voluntarily and as part of their rescue training varies greatly. This is the first paper, to the authors' knowledge, that documents the physical capabilities of mines rescue personnel.

The aerobic capacity of the mines rescue personnel (Table 2) was on average lower [7], similar [8-10], or higher [11,12] than other reported values for individuals working in mining operations around the world. The discrepancy between studies could be accounted for by the number of subjects evaluated, ranging from 18 [8] to 690 [10], and the methodology employed, with both "gold-standard" indirect calorimetry [9,11] and submaximal estimations from heart rate [7,8,10] being utilised to determine aerobic capacity. In comparison to other emergency response occupations, the average achieved by the mines rescue personnel was similar to the minimum aerobic capacity required to undertake the demands of fire fighting reported to be between 41 – 45 ml/kg/min [13-17], but significantly less than that expected of the Australian Federal Police (20 – 29 years: >51 ml/kg/min; 30–39 years: >42 ml/kg/min) [18], which corresponds to the 75^th ^percentile for the general Australian population. When compared against large international population based data from The Cooper Institute's Aerobics Center Longitudinal Study 1972–2002 [19], the maximal aerobic capacities of the mines rescue personnel lie in the 30–40^th ^percentile for the 20–29 and 40–49 age ranges, and the 50–60^th ^percentile for the 30–39 year olds.

Musculoskeletal endurance is a requirement of many emergency response situations where continuous displays of strength may be required. The results for the lower back endurance (Biering-Sorensen) test (Table 2) are similar to those achieved in another group of Australian coal miners [4]. Interestingly, both results are below normative values from sedentary populations [20]. The lower than expected scores obtained by the mining groups have been explained by repeated occupational associated microtrauma, causing muscular atrophy and weakness [21-23]. The cumulative effect of which may result in the functional deficits observed during testing.

Isometric strength has also been shown to be a valid predictor of endurance capabilities in mining [24]. The isometric strength tests, conducted in this study, assessed predominantly upper body musculature, with the exception of the deadlift that activates the majority of muscles in the torso, along with the quadriceps, hamstrings, and gluteus maximus. In comparison to the lower body, isometric strength capabilities in the upper body remain relatively unchanged up to the age of 50 years [25]. This is consistent with the current study (Table 2), however insufficient numbers in the 50–59 year age group and the large variability within age groups prevented any statistically significant findings.

The simulated work tasks were developed from task analyses and subsequently validated, by underground miners, for both their realism and physical demand [6]. The intensity of all the tasks was extremely demanding with heart rate responses averaging greater than 88% of age predicted maximal heart rates (Table 3), values similar to those recorded during fire fighter simulation protocols [26,27], and indicating that the rescue personnel were exerting near maximal effort throughout the tasks. The hose drag has been reported, by underground miners, to be physically more demanding than either the incremental carry or coal shovel [6]. However the heart rate responses recorded during the hose drag and coal shovel tasks were not significantly different (Table 3) and therefore may not be as discriminating as the recovery heart rate responses (Figure 1) in reflecting the physical demands of the tasks. Heart rate recovery following activity is correspondingly faster in those individuals who have a higher aerobic capacity [28-31].

The battery of tests, both general-health and task-related, provide an appropriate framework for the physical assessment of mines rescue personnel. The multitude of scenarios that a mines rescue team may experience require personnel to have a combination of both aerobic and muscular endurance, and absolute strength that will enable them to perform without excessive fatigue impairing their judgement and thus placing themselves and other members of their team at an increased risk of injury.

## Conclusion/recommendation

Mines rescue requires strenuous effort at sporadic intervals, and it is unlikely that the physical demands of work and the process of on the job rescues will be of sufficient frequency to provide adequate training to maintain, let alone increase, physical fitness. It is therefore recommended that (1) standards of required physical fitness be developed and (2) mines rescue personnel undergo regularly training (and assessment) in order to maintain these standards.

## Competing interests

The authors declare that they have no competing interests.

## Authors' contributions

IBS contributed to the study design, acquisition of data, analysis and interpretation of data, and drafted the manuscript. MDM contributed to the study design, acquisition of data, analysis and interpretation of data, and revision of the manuscript. APH contributed to the analysis and interpretation of data, and drafted the manuscript. TWP contributed to the study conception and design, and the revision of the manuscript. All authors read and approved the final manuscript.
